# Bayesian Optimization of Spray Parameters for the Deposition of Ga_2_O_3_–Cu_2_O Heterojunctions

**DOI:** 10.1021/acsaem.4c03284

**Published:** 2025-03-24

**Authors:** Maximilian Wolf, Georg K. H. Madsen, Theodoros Dimopoulos

**Affiliations:** a Institute of Materials Chemistry, TU Wien, Vienna 1060, Austria; b Center for Energy, AIT Austrian Institute of Technology GmbH, Vienna 1210, Austria

**Keywords:** machine learning, ultrasonic spray pyrolysis, thin film materials, optoelectronic devices, process parameter optimization, data subset selection, model evaluation

## Abstract

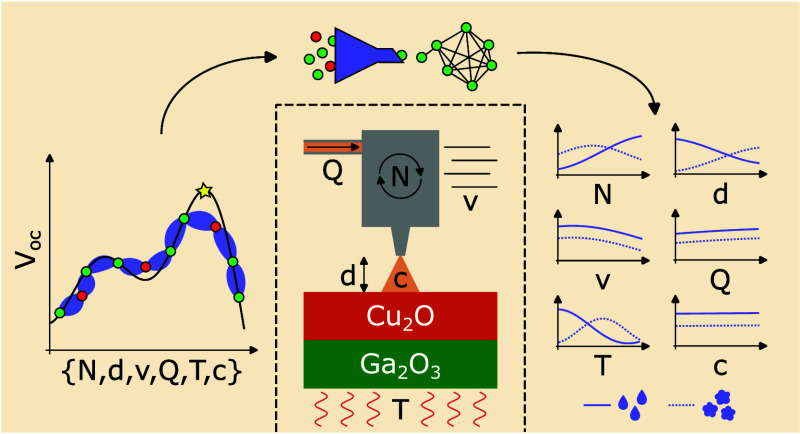

The accelerated discovery and optimization of materials relies on the integration of advanced experimental techniques with data-driven methodologies. In this work, Bayesian optimization (BO) is applied to optimize the ultrasonic spray pyrolysis (USP) process for the deposition of copper oxides, targeting high-quality Ga_2_O_3_–Cu_2_O heterojunctions for optoelectronic applications. By employing BO with an initial data set of 12 samples and conducting 4 USP parameter optimization cycles, significant improvements in device performance are achieved, with the open-circuit voltage increasing from 288 to 804 mV. During the optimization process, the performance of the model declines, necessitating the identification of a reliable subset of samples from the full data set. Through the application of BO, the cross-validation error of the model is minimized based on the sample selection, whereby accuracy is restored and generalizability is achieved. The subsequent model evaluation reveals two distinct deposition regimes, each characterized by unique process conditions, leading to specific material properties and device performances. These findings not only demonstrate the application of a data-driven experimental workflow in the context of thin film deposition but also highlight the importance of robust data validation and model evaluation.

## Introduction

Tackling the global challenge of transitioning to sustainable energy systems relies heavily on materials discovery and optimization. To accelerate this process, self-driving laboratories are emerging as solutions that integrate experiment automation and machine learning.^1−4^

In this context, Bayesian optimization (BO) has proven useful for exploring and optimizing material properties within high-dimensional parameter spaces. The method is based on the training of surrogate models, e.g., Gaussian processes (GP), to predict probability distributions, which enables efficient guidance of experimental efforts.^5,6^ In comparison to more traditional grid or random searches, BO can find global optima in fewer evaluation steps and delivers robust predictions by modeling the uncertainty. Even though there is a substantial and growing body of literature about the application of BO in material science, most studies focus on computational problems while examples for physical experiments are less common.^7^ Especially in the field of optoelectronic heterojunctions only a few case studies are reported.^8−12^

Ultrasonic spray pyrolysis (USP) is a well-established method for the deposition of optoelectronic thin films and has great potential for accelerating material discovery.^13−15^ Depending on the system, several process parameters can be tuned and provide high flexibility. The deposition mechanisms span the liquid, gas, and solid phases, where droplets directly wet the substrate, precursor vapors condense, and particles adhere to the surface, respectively.^16^ However, this flexibility comes at a price: small adjustments of process parameters can influence which mechanism occurs, making it difficult to find the optimum conditions. It is therefore a prime candidate for integrating BO to optimize the deposition of thin films.

In this case study, BO is applied for the USP deposition of Cu oxides, targeting high-quality Ga_2_O_3_–Cu_2_O n-p-heterojunctions. Both materials hold promising properties for the application in high-performance optoelectronic devices as electron and hole transport layers, respectively. The favorable energy band alignment between the two materials was shown to lead to high open circuit voltage values in solar cell devices. In a first report, electrochemically deposited Cu_2_O was combined with Ga_2_O_3_ prepared by atomic layer deposition,^17^ while in the second, sputtered Cu_2_O was combined with Ga_2_O_3_ deposited by USP.^18^ Water-based USP recipes for these materials were already reported by the present group,^19,20^ however, preliminary experiments of combining them in all-USP-fabricated heterojunction devices yield unsatisfactory results. Since the Cu_2_O deposition was developed on plain glass substrates and the Ga_2_O_3_ film probably leads to changes in the surface and thermal properties as well as to chemical interactions, the deposition recipe for copper oxide needs to be tuned.

The research structure of this work consists of three main aspects: (i) parameter optimization of the copper oxide deposition to increase the open circuit voltage of the heterojunction device, (ii) model optimization to improve the predictive strength of the surrogate model by selecting the data subset which maximizes the error of the leave-one-out cross-validation, and (iii) parameter evaluation to analyze the established dependencies and reveal the underlying deposition mechanisms. Finally, the optimized model is validated by additional deposition experiments which unfolds the risks of applying BO on exposed research equipment and facilitates the identification of a faulty system component.

## Experimental Section

### Sample Preparation

ITO glass substrates with a size of 25 mm × 25 mm (Ossila Ltd.) are cleaned by progressive ultrasonication at 50 °C for 15 min in 1 vol % Hellmanex III washing solution (Hellma GmbH & Co. KG), deionized water, and isopropanol (Carl Roth GmbH & Co. KG, 9866). The dried substrates are placed on the hot plate of the USP system (Sono-Tek Corp., ExactaCoat) which is equipped with a 120 kHz ultrasonic nozzle (Sono-Tek Corp., Impact), operating at 3.5 W power.

For the deposition of a ∼ 20 nm thick Ga_2_O_3_ film,^19^ the hot plate is heated to 380 °C and a 40 mM gallium(III) acetylacetonate (Sigma-Aldrich, 393541) water-based solution with 20 vol % acetic acid (Sigma-Aldrich, A6283) is deposited in 50 cycles at a nozzle distance *d* of 200 mm, nozzle speed *v* of 167 mm min^–1^, and solution flow rate *Q* of 0.8 mL min^–1^.

Subsequently, the deposition of copper oxides is carried out using a solution of 8 vol % acetic acid in deionized water with a copper(II) acetate monohydrate (Sigma-Aldrich, 229601) concentration *c* and constant copper-to-glucose (Sigma-Aldrich, G7528) molar ratio of 1:2.^20,21^ For each individual sample, the hot plate temperature *T*, the number of deposition cycles *N* and the other process parameters *d*, *v*, *Q*, and *c* are different.

The heterojunction devices are finished by scratching the sample corners with a diamond cutter to expose the underlying ITO, followed by sputtering a 200 nm thick gold layer through a mask with an 8 × 8 array of 2.54 mm^2^ large holes.

This produces 64 individual devices in a single sample preparation workflow, facilitating the acquisition of statistically robust photovoltaic performance data and enabling to investigate its spatial distribution over the whole sample surface.

### Crystal Phase Quantification

Prior to sputtering the gold contacts, X-ray diffraction (XRD) patterns are recorded under ambient conditions using a grazing incidence XRD instrument (Thermo Fisher Scientific Inc., ARL Equinox 100) with Cu–K_α_ radiation directed at the surface at an angle of 5°.

Depending on the process parameters of the copper oxide deposition, combinations of Cu_2_O (COD^22^ 96–900–5770), CuO (COD 96–101–1149), and Cu (COD 96–710–1265) can be identified. The ITO coating of the substrate is measured as In_2_O_3_ (COD 96–231–0010) with varying intensity but the small amount of nanocrystalline Ga_2_O_3_ is not detectable.

For the quantification of the phases, Rietveld refinement is carried out as implemented in FullProf^23^ and interfaced by Match!^24^ phase analysis software. A systematic refinement procedure is followed to ensure that the quantification results are consistent within this study (S1).

### Current–Voltage Characteristics

The electronic properties of the heterojunctions are determined by measuring the current–voltage characteristics (IVs) with a semiconductor parameter analyzer (Agilent 4156C) in the dark and under simulated solar illumination (AM1.5G, Ossila Solar Simulator G2009). Automatized measurement of the 8 × 8 device array on each sample is carried out using the MA8 × 8 contact interface and related software of the COHESIVM package.^25^

For the identification of shunted and abnormally behaving devices, the dark IVs are fitted against a single diode model to determine the shunt resistance *R*_*sh*_, the ideality factor *n*, and the coefficient of determination *R*^2^ (S2). If one of the conditions *R*_*sh*_ < 10 kΩ, *n* < 2, or *R*^2^ < 0.9 is met, the measurement is removed from the data set.

Finally, photovoltaic performance parameters are extracted from the light IVs using the methods provided by COHESIVM. For this study, only the open circuit voltage *V*_*oc*_ and the short circuit current density *J*_*sc*_ are considered because they are representative for the quality of the heterojunctions. The measurements are filtered again if the *V*_*oc*_ falls outside the interval [0.0, 1.0] V or if *J*_*sc*_ > 0.

## Results and Discussion

### Initial Data Set

Latin hypercube sampling (LHS) as provided by NUBO^26^ in version 1.2.0 is employed to efficiently cover the six-dimensional USP parameter space of the copper oxide deposition (S3) in 11 unique parameter combinations. As mentioned in the introduction, the base recipe (BR) corresponds to previously established Cu_2_O deposition parameters that yield suboptimal results when applied to Ga_2_O_3_. Together with this BR, the initial data set contains 2 samples for each parameter (Table S4). This small amount of data is deliberately chosen such that the approach is more efficient than a conventional design of experiment, where 3–5 parameter variations are common.

The phase compositions of the copper oxide films are distributed between 0% and 100% Cu_2_O fraction with various thicknesses, indicated by the measured amount of In_2_O_3_ (Figure S3). As mentioned in the introduction, the base recipe does not work for the deposition of pure Ga_2_O_3_–Cu_2_O heterojunctions which is evidenced here by the detection of CuO (Table S5).

After evaluating and filtering the IVs based on the criteria stated in the experimental section, the photovoltaic performance metrics of a total of 644 individual data points can be used for the training of the initial model (Table S6). Among them, the largest values over the 8 × 8 device array and  are 783 mV and −1.21 mA cm^–2^, respectively, which is already a significant improvement over the BR sample (469 mV, −0.68 mA cm^–2^). However, the largest average values *V*_*oc*_^8*x*8^ and *J*_*sc*_^8*x*8^ are 517 mV and −0.96 mA cm^–2^, respectively, motivating the optimization of the sample homogeneity.

### Surrogate Model

To model the dependencies between the process parameters and the heterojunction quality, GPyTorch^27^ in version 1.11 is employed for the training of a GP. To this end, the built-in Matérn kernel with a smoothness parameter ν = 2.5 and automatic relevance determination of each input parameter is adopted.^5,28^ The covariance function is modified by adding a trainable nugget term over the diagonal of the covariance matrix^29^ and the output is scaled by another trainable parameter. The predictions are modeled as a Gaussian distribution from which the marginal log likelihood is calculated and maximized during the training. This loss function is optimized using Adam^30^ as implemented in PyTorch^31^ in version 2.2.1 with a scheduler that reduces the learning rate on plateaus from its initial value of 0.2 by 10% with a patience of 50 iterations.

The applicability of the model architecture and training procedure is verified by three experiments of predicting the *V*_*oc*_ from the USP process parameters. The root mean squared error (RMSE) between the normalized true target values *y*_*norm*_ and target predictions *ŷ*_*norm*_ is used as performance metric. (i) A random split of the initial data set into 90% training and 10% test data, where the input parameters are augmented by the positions of the individual data points on the 8 × 8 device array. (ii) A leave-one-out cross-validation (LOOCV), where the data points are aggregated on a per-sample basis. The model is trained on the data of all but one sample and the prediction of the left-out sample is evaluated. This process is repeated for every sample and the errors are combined in a single RMSE. (iii) The LOOCV is additionally applied to the *V*_*oc*_^8*x*8^ data without input parameter augmentation.

**Figure 1:**
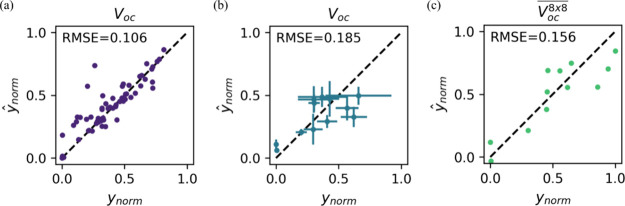
Results of the surrogate model verification based on the prediction of the *V*_*oc*_ from the USP process parameters in the initial data set. (a) Random training-test split of individual data points. (b) LOOCV of sample-wise aggregated data points. The mean of the predictions and their standard deviation is displayed. (c) LOOCV of sample-wise averaged data points.

The results of the experiments (Figure 1) verify that the model (i) has the capacity to map process parameters to photovoltaic performance metrics, (ii) generalizes decently within the parameter space, and (iii) is also applicable for statistically derived values.

### Parameter Optimization

A major goal of this study is to find the USP process parameters of the copper oxide deposition which maximize the quality of the Ga_2_O_3_–Cu_2_O heterojunction. For quantifying this quality, the *V*_*oc*_ alone is selected to act as a proxy because, at otherwise consistent device architecture, it is mainly influenced by the properties of the copper oxide film and its interface with Ga_2_O_3_. The *J*_*sc*_, on the other hand, is highly dependent on the film thickness and including it would add another layer of complexity to the parameter optimization (PO) problem.

However, motivated by the condition of the initial data set, the homogeneity is addressed as well to end up with a spatially uniform deposition process. To this end, the GP is restructured into a multitask variant with cross-talk between the covariances of each target as implemented in GPyTorch^27^ in version 1.11. This allows to simultaneously model the *V*_*oc*_^6*x*6^ and *V*_*oc,max*_^6*x*6^, enabling to describe a homogeneous maximum while benefiting from the covariance cross-talk (Figure S4) as expected.^32^ Modeling the standard deviation significantly worsens the performance (Figure S5), and directly training on individual data points to derive the statistics from the predictions is considered inapplicable because it would require a very good model of the spatial noise distribution. The 6 × 6 inner array of devices is used to reduce the influence of edge effects which is supported by an increase of the model performance over using the whole 8 × 8 device array (Figure S6).

PO is carried out using NUBO^26^ in version 1.2.0 in the constrained multipoint optimization setting with the USP parameter space (S3) as boundaries and a sequential batch size of 4. This facilitates the integration into the manual workflow where the preparation of 4 samples is most efficient. The acquisition function is a modification of the implemented Monte Carlo upper confidence bound (MCUCB)^33,34^ which enables the use of the multitask GP. Instead of sampling the distribution of a single target, the new function samples from the GPyTorch multitask multivariate normal distribution and aggregates the targets in a figure of merit (FoM) before calculating the result. This approach has the advantage that the model can still benefit from the covariance cross-talk while the other parts of the optimization procedure remain unchanged. Directly training the model with the FoM has a lower performance than the multitask version (Figure S7), probably because some information is lost when the FoM is calculated. The MCUCB is parametrized with a trade-off parameter  = 1.96, corresponding to the 95% confidence interval, and a sampling size of 512. Optimization of the acquisition function is performed in 10 random starts using Adam^30^ with a fixed learning rate of 0.1.

**Figure 2:**
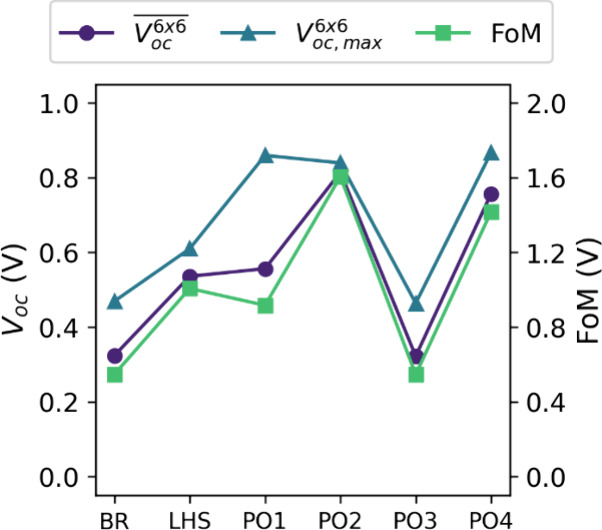
PO metrics for the samples with the highest FoM in each data set slice (BR: base recipe, LHS: Latin hypercube sampling, POn: n^th^ parameter optimization cycle).

The FoM is empirically compiled by computing different algebraic configurations of *V*_*oc*_^6*x*6^ and *V*_*oc,max*_^6*x*6^. The configuration which best corresponds to the manual ranking of the *V*_*oc*_ maps (S10) is selected. Mathematically, the expression penalizes samples which have a high *V*_*oc,max*_^6*x*6^ in comparison to their *V*_*oc*_^6*x*6^:
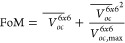


A total of 4 PO cycles (PO1, PO2, PO3, and PO4) is carried out and yields an increase of the FoM by 61% over the initial data set already in PO2 (Figure 2). The optimized sample has the largest *V*_*oc*_^6*x*6^ in the whole data set (Table S6) with 815 mV which is over two times as high as for the BR sample and almost 50% larger than the highest value in the LHS data set. The largest *V*_*oc,max*_^6*x*6^ of 867 mV is obtained in PO4 with a sample that still has a very high FoM. In the context of BO, the low values for PO3 can be interpreted as an exclusive exploration cycle.

However, the *J*_*sc*_ is much smaller in the optimized samples by almost a factor of 100 in comparison to the largest values in the initial data set (Table S6). Admittedly, this photovoltaic performance metric is deliberately excluded from the PO but such a significant change cannot be explained by thickness fluctuations of the copper oxide film alone and is rather the consequence of current blocking defects. These defects could originate from the incomplete oxidation of the copper oxide film, resulting in elemental Cu (Table S5). Moreover, the performance of the multitask GP model significantly decreases during the PO (Figure S8). Declining predictive accuracy is not uncommon in BO because new data points are added locally which leads to less-uniform input parameter distribution and poorer generalizability. However, the drastic increase of the LOOCV error between PO1 and PO2 indicates increasing problem complexity or, more likely, inconsistencies of the USP deposition conditions, which are also discussed later. Even though robust BO approaches exist,^6,35^ they were deemed inapplicable from the beginning of this case study because they inherently require larger data sets. Since the process conditions which yield the highest FoM lead to an unexpectedly low *J*_*sc*_ and the collected data is suspected to contain inconsistent results, the PO is concluded after four optimization cycles.

### Model Optimization

To recover the predictive strength of the multitask GP architecture and enable the exploitation of the collected data, the model is optimized in terms of data subset selection. The Ax platform^36^ in version 0.4.0 is used for the BO of the sample selection because it provides constrained outputs and efficient handling of a large number of discrete inputs. The targets of the GP are changed to *V*_*oc*_ and *J*_*sc*_ which requires the use of the position augmented individual data points but provides already improved performance over the final PO model (Figure S9). The objective of the model optimization (MO) is to find the subset of samples which minimizes the RMSE of the LOOCV while the range of the *V*_*oc*_ and *J*_*sc*_ within this subset must be larger than 0.7 V and 4.0 log(mA cm^–2^), respectively. Additionally, the minimum number of samples (MNS) which are included in the model is enforced in three different scenarios with an MNS of 10, 15, and 20. Each scenario is started twice with the default generation strategy of the Ax platform and the BO is continued until the model runs into numerical issues where the process terminates.

**Figure 3:**
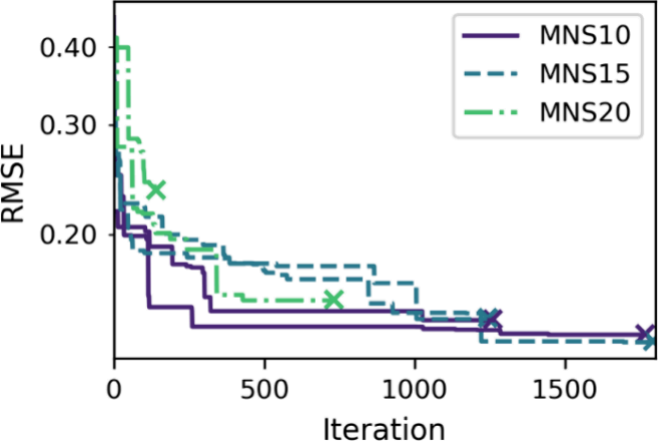
Optimization trace of the model optimization to minimize the RMSE of the LOOCV. Each MNS (minimum number of samples) case is started twice and terminates where the line ends with a cross.

The best run corresponds to the cases with a lower sample limit of 15 (MNS15) and terminates after almost 1800 iterations but achieves the lowest RMSE only about 100 iterations earlier (Figure 3). The optimized subset consists of 19 samples and the selection of individual samples is consistent within at least two other runs (Table S7).

In comparison to the model which is trained on the complete data set (Figure S9), the optimized model has a 26% and 17% lower LOOCV RMSE for predicting the *V*_*oc*_ and *J*_*sc*_, respectively (Figure S10). Additionally, the model performs better than when the same architecture is trained on the initial data set (Figure S11), while covering a larger fraction of the process parameter space and larger ranges of the photovoltaic performance metrics. Even though the previous targets are not included in the MO, the performance of predicting the *V*_*oc*_^6*x*6^ and *V*_*oc,max*_^6*x*6^ is increased as well (Figure S12). Finally, following the exact same GP training procedure as before but for the prediction of the Cu_2_O fraction from the crystal phase quantification, the LOOCV RMSE is cut in half by using the optimized sample subset instead of the whole data set (Figure S13). These results confirm the effectiveness of the MO procedure and support the assumption that a generalized model is found.

### Model Evaluation

The optimized model is exploited by searching the process parameters which lead to extrema in the *V*_*oc*_^8*x*8^ and *J*_*sc*_^8*x*8^ surfaces. For full control over the procedure, a custom loss function is implemented which predicts the 8 × 8 device array, calculates its mean, and penalizes out-of-bounds input values. Like in the training of the surrogate model, Adam is used to optimize the loss which is minimized or maximized to find troughs *t* or peaks *p*, respectively. LHS, as already used for the initial data set, is employed to generate a quasi-random set of 30 process parameter combinations. The optimization procedure is run with each of these 30 distinct starting values until the loss converges with a tolerance of 0.1‰.

As a result, six extrema are found of which two maximize the *V*_*oc*_^8*x*8^ (Table S8) and one maximizes the *J*_*sc*_^8*x*8^ (Table S9). However, the input vectors of (*MO*) and (*MO*) are very similar, indicating that this set of process parameters corresponds to the deposition of a copper oxide film with a well-balanced trade-off between the photovoltaic performance metrics. For each of the three maxima, the individual process parameter dependencies on *V*_*oc*_ and *J*_*sc*_ are simulated. To this end, 100 device array means are predicted for each process parameter inside the lower and upper boundaries while keeping all other parameters constant.

The whole model evaluation (ME) procedure is repeated for the model which is trained on the initial data set. Here, one maximum for each of the photovoltaic performance parameters is found (Table S10, Table S11).

**Figure 4:**
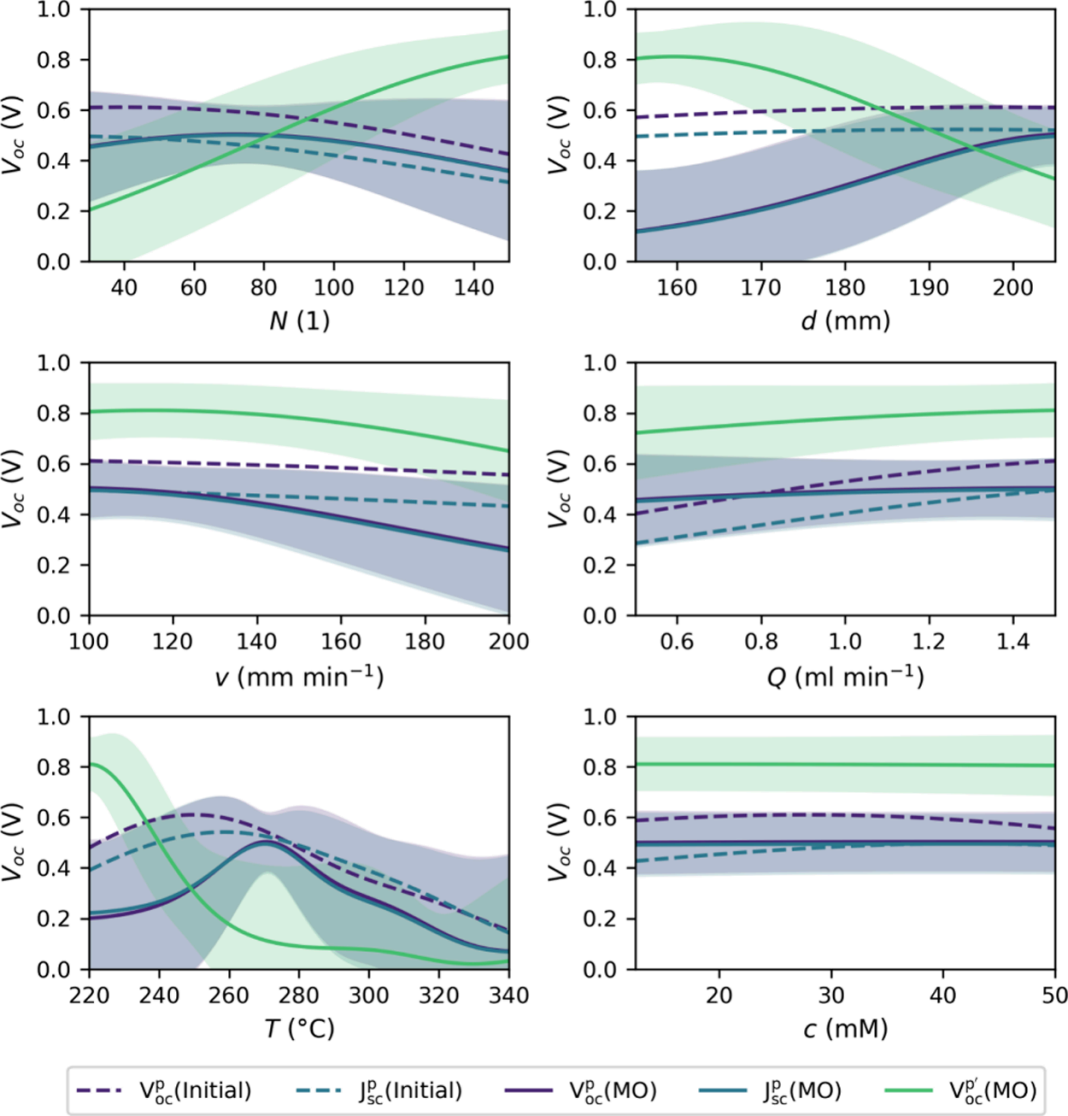
Predicted process parameter dependencies on the *V*_*oc*_ corresponding to the sets of parameters which maximize the *V*_*oc*_ and *J*_*sc*_ in the models trained on the initial data set and after the model optimization (MO). The shaded areas are the confidence regions of two times the standard deviation of the respective predictions.

As shown in Figure 4, the *V*_*oc*_ dependencies from the initial-data set-model are relatively flat with variation below 0.2 V overt the whole parameter range, except for *T*. In general, they agree with the *V*_*oc*_^*p*^(*MO*) and *J*_*sc*_^*p*^(*MO*) curves within their confidence region and only *d* is predicted to behave in a significantly different manner. However, in the MO-data set-model, *v* is a little more important, whereas the dependencies of *Q* and *c* are almost negligible. Additionally, the peak of *T* is shifted to higher values.

The *V*_*oc*_^*p*^′^^(*MO*) curves, on the other hand, completely differ from the other *V*_*oc*_ dependencies. While *v*, *Q*, and *c* are only shifted by a constant amount, the remaining process parameters exhibit a more complex behavior. Consequently, it can be assumed that there are at least two distinct processes with independent parameter requirements, probably leading to the two distinct *V*_*oc*_^8*x*8^ maxima in the optimized model (Table S8). The process related to the *V*_*oc*_^*p*^(*MO*) curves is characterized by a higher hot-plate temperature and favors a large nozzle distance. These parameters correspond to a process that requires sufficient evaporation of the precursor solution, indicating controlled growth from the gas phase. This is also supported by *N* where a limit is reached after which further deposition cycles lead to decreasing performance, suggesting a change in the growth mechanism. The *V*_*oc*_ dependencies from the *V*_*oc*_^*p*^′^^(*MO*) parameters, however, illustrate a different picture. For the related process, *T* and *d* must be low, corresponding to less evaporation and growth predominantly from the liquid phase. In this case, a higher number of deposition cycles progressively covers the surface more uniformly, potentially filling gaps and improving film continuity.

For the prediction of *J*_*sc*_, almost all parameter cases show similar tendencies (Figure S14). A significant exception are the *d* dependencies of the initial-data set-model which, otherwise, also agree in terms of magnitude with the *V*_*oc*_^*p*^(*MO*) and *J*_*sc*_^*p*^(*MO*) curves. Most importantly, the *V*_*oc*_^*p*^′^^(*MO*) parameters correspond to considerably worse *J*_*sc*_, solidifying the assumption of a less-controlled deposition process that apparently leads to current-blocking defects.

### Model Validation

The final measure of this case study is the validation of the predictive strength of the model through the preparation of two additional samples. The first one is prepared using the mean of the (*MO*) and (*MO*) parameters, and the second one is prepared using the (*MO*) parameters, denoted as ME1 and ME2, respectively.

Even though very similar process parameters are already tested within the initial LHS and subsequent PO cycles, the previous properties are not achieved (S4). ME1 resembles what would be expected from process parameters similar to *V*_*oc*_^*p*^′^^(*MO*) which produce Cu-containing samples with a high FoM but low *J*_*sc*_^8*x*8^. ME2, on the other hand, has almost completely blocked current and could not be measured with the employed equipment. Therefore, the results rather serve as demonstration of the reproducibility issues with the USP system which probably is the origin of the model performance degradation during the PO as well.

Indeed, since the properties of ME1 correspond to a liquid-phase deposition process, the problem must be related to incomplete evaporation. In this context, the most important system components are (i) the hot plate which supplies the required heat energy, and (ii) the ultrasonic nozzle which produces small enough droplets. The former component was readily excluded from the possible candidates by checking the temperature controller with an external temperature probe. However, the microstructure images of two Ga_2_O_3_ films, one prepared before and the second prepared after the problem, support the assumption of insufficient nebulization (Figure S15). Beyond this, the ultrasonic nozzle was dismantled and reassembled between the PO and ME which probably is the root cause of the issue.

Finally, to further solidify this assumption and pursue a conclusive model validation (MV), the influence of *c* is investigated. Reducing the precursor solution concentration can have an effect on the droplet size^37,38^ and, naturally, decreases the thermal energy needed for the decomposition of the precursor species. Consequently, the evaporation can run more efficiently and the deposition from the gas phase could be restored. To this end, the deposition of ME1 is repeated with 50% and 25% of the initial *c* to produce MV1 and MV2, respectively (Table S4).

**Figure 5:**
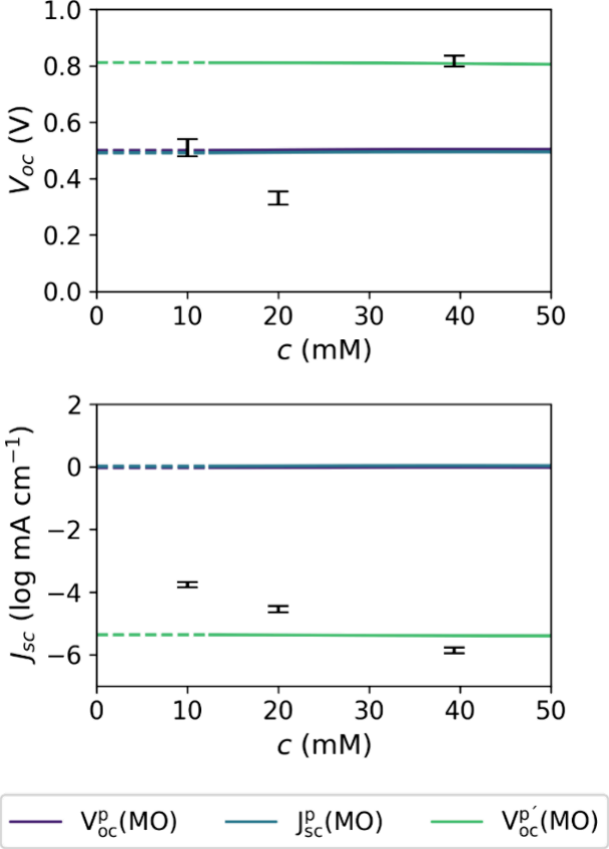
Photovoltaic performance metrics of ME1, MV1, and MV2, which have *c* values of 39.4, 20.0, and 10.0 mM, respectively, displayed as error bars of the 95% confidence interval over the 8 × 8 device array. The solid lines are the predicted dependencies from the ME, and the dashed lines are their constant extrapolations.

The results of the three samples in Figure 5 align with the previously stated hypothesis. Through the reduction of *c*, the deposition process seemingly transitions from the liquid-phase to the gas-phase regime. After an intermediary minimum which could be the result of a mixed-regime deposition, the *V*_*oc*_ lands exactly at the value which is predicted in the ME. These results apparently contradict the previously established *c*-independence of the *V*_*oc*_ (Figure 4) but the model presumably only works for a fixed deposition regime. This nonlinear behavior at the boundary of different growth processes could be an additional contribution to the model performance degradation during the PO. The *J*_*sc*_ also approaches the predicted value but ends up at a much smaller value. However, since the USP deposition of the Ga_2_O_3_ film also suffers from the increased droplet size (Figure S15), regions with higher film thickness could block the current and lead to a smaller effective area.

## Conclusions

Bayesian optimization (BO) is successfully applied with an initial data set of 12 samples and 4 optimization cycles of each 4 samples, to find the process parameters for the ultrasonic spray deposition of copper oxides which maximize the *V*_*oc*_ of heterojunctions with Ga_2_O_3_. As a result, the average and maximum *V*_*oc*_ on samples with 8 × 8 individual devices increase from 288 mV and 469 mV to 804 mV and 856 mV, respectively.

Since erroneous data is suspected to be responsible for a drastic decrease of the surrogate model accuracy, the subset of the samples which yield the lowest error in the cross-validation is searched, again through the application of BO. The optimized subset includes 19 of total 28 samples, results in a model which has a better performance than the initial model prior to the parameter optimization, and generalizes well over different model targets.

Through the model evaluation with respect to the optimized process parameters, two distinct deposition regimes – liquid-phase and gas-phase growth – can be identified. The former one is characterized by a low hot-plate temperature *T*, short nozzle-to-substrate distance *d*, and large number of deposition cycles *N*, leading to a high *V*_*oc*_ over 800 mV but blocked current due to the formation of elemental Cu. The latter requires a higher *T*, long *d*, and lower *N* to form pure Cu_2_O with a lower *V*_*oc*_ around 500 mV but over 100 times larger *J*_*sc*_.

Finally, the model validation is impaired by a defective ultrasonic nozzle which results in less efficient nebulization and larger droplets during the USP deposition. Despite this, the established process parameter dependencies facilitate the identification of this problem and enable the development of a strategy to restore the desired deposition conditions.

## Supporting Information Available

Data tables and figures that are referenced in the main text (PDF)
CSV files of the complete data that is extracted from IV and XRD measurements and used for the training of the models (ZIP)

## Notes

**Code Availability:** The utilized Python code for the Gaussian process training, Bayesian optimization, and model evaluation is archived under 10.5281/zenodo.14913980.
