# Supplementary material for: Bayesian Optimization of Spray Parameters for the Deposition of Ga2O3–Cu2O Heterojunctions
Source: ACS Appl Energy Mater. 2025 Mar 24;8(7):4362–9. doi: 10.1021/acsaem.4c03284 (PMC12001286; doi:10.1021/acsaem.4c03284)
Supplement: Supplementary file 1 — ae4c03284_si_001.pdf [file ae4c03284_si_001.pdf]

Supporting Information

Bayesian optimization of spray parameters for the deposition of Ga_2O_3-Cu_2O heterojunctions

[a,b,*]*Maximilian Wolf,* [b]*Georg K. H. Madsen,* [a,+]*Theodoros Dimopoulos*

[a] Center for Energy, AIT Austrian Institute of Technology GmbH, Austria.

[b] Institute of Materials Chemistry, TU Wien, Austria.

[*] maximilian.wolf@ait.ac.at, [+] theodoros.dimopoulos@ait.ac.at

Table of Contents

Table S1. Symbols and abbreviations which are recurrently used in this work, sorted by their first appearance in the main text......4

S1 Rietveld Refinement 6

S2 Single Diode Model Fitting 7

Table S2. Symbols in the single diode model......7

Figure S1. Estimation of starting values for the fitting of the measurement against the single diode model. (a) Linear fit of the shunt current for the estimation of Rsh. (b) Programmatic search for linear measurement sections which intercept at the tun-on voltage. (c) Linear fit of the exponential current for the estimation of n and I0. 8

Figure S2. Result of fitting a measurement against the single diode model in a normal (left) and semi-logarithmic (right) plot. 8

S3 Parameter Space 9

Table S3. Considered parameter ranges for the USP deposition of copper oxides......9

S4 Sample Data Overview 10

Table S4. Process parameters for the USP deposition of copper oxides. 10

Table S5. Phase composition and texture parameters from the Rietveld refinement of the XRD measurements. ..11

Figure S3. Ternary phase diagram with the data from Table S5, grouped into their process parameter origin. The occasionally measured elemental Cu is added to the amount of Cu_2O. 12

Table S6. Photovoltaic performance metrics......13

S5 Effect of GP Target Selection 14

Figure S4. LOOCV of the surrogate model trained on the initial dataset for the prediction of (a) the average and (b) the maximum Voc over the 6 × 6 inner array of devices, and (c) the multitask prediction of both values simultaneously......14

Figure S5. LOOCV of the surrogate model trained on the initial dataset for the multitask prediction of the average Voc and its standard deviation over the 6 × 6 inner array of devices. 14

Figure S6. LOOCV of the surrogate model trained on the initial dataset for the multitask prediction of the average and the maximum Voc over the 8 × 8 array of devices. 14

Figure S7. LOOCV of the surrogate model trained on the initial dataset for the prediction of the FoM in the 6 × 6 inner array of devices. 15

S6 Model Degradation during PO 16

Figure S8. LOOCV of the surrogate model for the multitask prediction of the average and the maximum Voc over the 6 × 6 inner array of devices, trained during the parameter optimization on an increasing number of datapoints through including samples from (a) PO1, (b) PO2, (c) PO3, and (d) PO4......16

S7 Results of Model Optimization 17

Table S7. Sample selection which yields the LOOCV with the lowest RMSE during the model optimization in 2 runs for each MNS of 10, 15, and 20 samples........ 17

Figure S9. LOOCV of the surrogate model trained on the complete data after the parameter optimization for the multitask prediction of the Voc and Jsc of individual datapoints. 18

Figure S10. LOOCV of the surrogate model trained on the optimized dataset after the model optimization for the multitask prediction of the Voc and Jsc of individual datapoints. 18

Figure S11. LOOCV of the surrogate model trained on the initial dataset for the multitask prediction of the Voc and Jsc of individual datapoints. 18

Figure S12. LOOCV of the surrogate model trained on the optimized dataset after the model optimization for the multitask prediction of the average and the maximum Voc over the 6 × 6 array of devices. 19

Figure S13. LOOCV of the surrogate model trained on (a) the complete dataset after the parameter optimization and (b) the optimized dataset after the model optimization for the prediction of the Cu_2O fraction........ 19

S8 Results of Model Evaluation 20

Table S8. Extrema of the average Voc over the 8 × 8 device array after model optimization........ 20

Table S9. Extrema of the average Jsc over the 8 × 8 device array after model optimization. 20

Table S10. Extrema of the average Voc over the 8 × 8 device array in the initial dataset........ 20

Table S11. Extrema of the average Jsc over the 8 × 8 device array in the initial dataset. 20

Figure S14. Predicted process parameter dependencies on the Jsc corresponding to the sets of parameters which maximize the Voc and Jsc in the models trained on the initial dataset and after the model optimization. The shaded areas are the confidence regions of the respective predictions........ 21

S9 Problem with Nebulization 22

Figure S15. Microstructure of the USP-deposited Ga_2O_3 which is used in this work, recorded by scanning electron microscopy, the insets show the same film at 10x the magnification. (a) Initially, a homogenous film with a few particles scattered around the surface is observed. (b) After the hypothesized nozzle failure, a lot of speckles are visible which appear like impacted droplets at higher magnification. 22

S10 Maps of Open Circuit Voltage 23

Table S12. Voc maps of all samples which show the measured open circuit voltage for each individual datapoint. The color palette is fixed to a range of [0, 1] V and the center value in the scale bar denotes the average Voc. 23

References 27

Table S1. Symbols and abbreviations which are recurrently used in this work, sorted by their first appearance in the main text.

Symbol	Meaning
BO	Bayesian optimization
GP	Gaussian process
USP	Ultrasonic spray pyrolysis
d	Nozzle-to-substrate distance
v	Nozzle movement speed
Q	Precursor solution flow rate
c	Copper(II) acetate monohydrate concentration
T	Hot plate temperature
N	Number of deposition cycles
XRD	X-ray diffraction
COD	Crystallography Open Database[1]
IV	Current-voltage characteristic
R_{sh}	Shunt resistance
n	Ideality factor
R^2	Coefficient of determination
V_{oc}	Open circuit voltage
J_{sc}	Short circuit current density
LHS	Latin hypercube sampling
BR	Base recipe
$V_{oc,max}^{8x8}$	Maximum V_{oc} over the 8 × 8 device array
$J_{sc,min}^{8x8}$	Minimum J_{sc} over the 8 × 8 device array
$\overline{V_{oc}^{8x8}}$	Average V_{oc} over the 8 × 8 device array
$\overline{J_{sc}^{8x8}}$	Average J_{sc} over the 8 × 8 device array
RMSE	Root mean squared error
y_{norm}	Normalized true target values
$\hat{y}_{norm}$	Normalized target predictions
LOOCV	Leave-one-out cross-validation
PO	Parameter optimization
$\overline{V_{oc}^{6x6}}$	Average V_{oc} over the 6 × 6 inner array of devices

$V_{oc,max}^{6x6}$	Maximum V_{oc} over the 6 × 6 inner array of devices
MCUCB	Monte Carlo upper confidence bound
FoM	Figure of merit
MO	Model optimization
MNS	Minimum number of samples
t	Trough
p	Peak
$V_{oc}^{p}(MO)$	First peak in the $\overline{V_{oc}^{8x8}}$ surface of the MO-dataset-model
$V_{oc}^{p'}(MO)$	Second peak in the $\overline{V_{oc}^{8x8}}$ surface of the MO-dataset-model
$J_{sc}^{p}(MO)$	Peak in the $\overline{J_{sc}^{8x8}}$ surface of the MO-dataset-model
ME	Model evaluation
$V_{oc}^{p}(Initial)$	Peak in the $\overline{V_{oc}^{8x8}}$ surface of the initial-dataset-model
$J_{sc}^{p}(Initial)$	Peak in the $\overline{J_{sc}^{8x8}}$ surface of the initial-dataset-model
MV	Model validation

S1 Rietveld Refinement

To obtain consistent phase quantification results from XRD measurements, a systematic approach is followed using the FullProf[2] backend which is interfaced by Match![3] phase analysis software:

(i) To begin with, the diffractograms are trimmed between 25° and 90° (no elemental Cu) or 100° (elemental Cu present) scattering angle. Then, the identified structures from the set of Cu_2O (COD 96-900-5770), CuO (COD 96-101-1149), Cu (COD 96-710-1265), and In_2O_3 (COD 96-231-0010) are loaded into the program.

(ii) The peak shapes are fixed to a Gaussian profile to account for the thin-film-induced peak broadening, except for In_2O_3 and large amounts of Cu_2O, CuO, and Cu, where a Lorentzian profile is used.

(iii) After identifying the involved crystal structures, the scale factors of all phases are fitted, consecutively followed by the zero-point shift of the profile, the first background parameter, and all independent unit cell parameters.

(iv) Preferred orientation of Cu_2O in $[111]$ direction[4,5] and CuO in $[\bar{1}11]$ direction[6,7] was modelled using the modified March texture function of the utilized refinement software.

(v) The parameters of the Caglioti FWHM as implemented in FullProf are fitted one after the other while also adjusting the scale factors.

(vi) Finally, the scale factors, all background parameters, and the preferred orientation values are fitted once more while keeping the other parameters constant.

S2 Single Diode Model Fitting

An automatized evaluation of the dark IV properties is carried out by fitting the measurements against a single diode model:

$$I = I_L - \frac{V + IR_s}{R_{sh}} - I_0 \left(\exp\left(\frac{V + IR_s}{nV_{th}} \right) - 1 \right)$$

Table S2. Symbols in the single diode model.

Symbol	Meaning
I	Current
I_0	Saturation current
I_L	Photocurrent
n	Ideality factor
R_s	Series resistance
R_{sh}	Shunt resistance
V	Voltage
V_{th}	Thermal voltage at 300 K

For reasonable starting values which facilitate the convergence of the fitting algorithm, the shunt current and the exponential current are approximated. The former is modelled using a linear fit in the interval of [-0.4, 0.4] V (Figure S1a), where the shunt resistance can be estimated:

$$I = \frac{V}{R_{sh}}$$

The latter enables to estimate the ideality factor as well as the saturation current from the linearized exponential term in which the series voltage is negligible:

$$\frac{1}{\ln I} = \ln I_0 + \frac{V}{nV_{th}}$$

Since the voltage region of this exponential behavior is not fixed, the forward voltage V_F is graphically located as first inflection point. The linear measurement sections which intercept at the inflection point are programmatically searched in the interval of [1.5, 3.5] V (Figure S1b). From this V_F fitting, the line with the larger slope is used for estimating n and I_0 (Figure S1c).

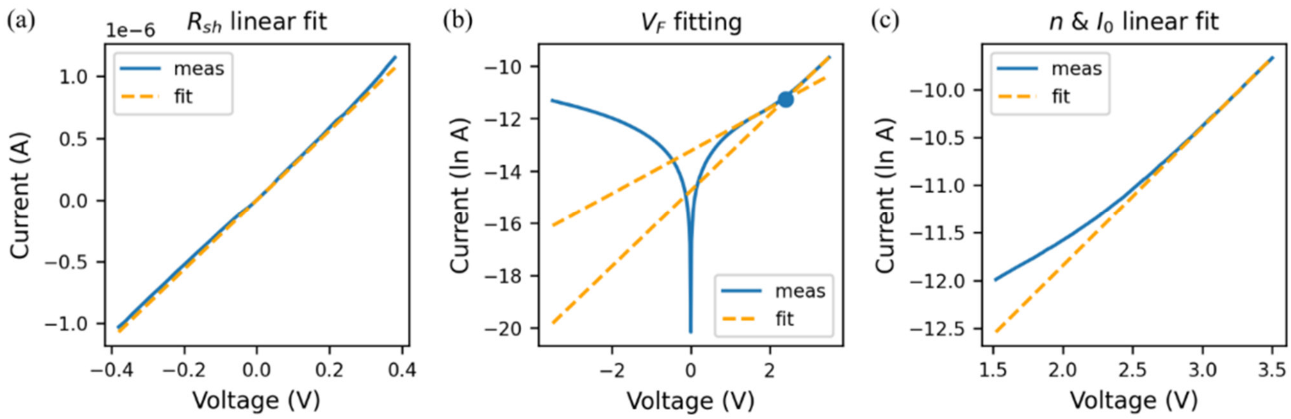

Figure S1. Estimation of starting values for the fitting of the measurement against the single diode model. (a) Linear fit of the shunt current for the estimation of R_{sh}. (b) Programmatic search for linear measurement sections which intercept at the tun-on voltage. (c) Linear fit of the exponential current for the estimation of n and I_0.

Subsequently, the whole measurement is fitted against an explicit solution of the single diode model[8] from which the coefficient of determination R^2 is calculated (Figure S2).

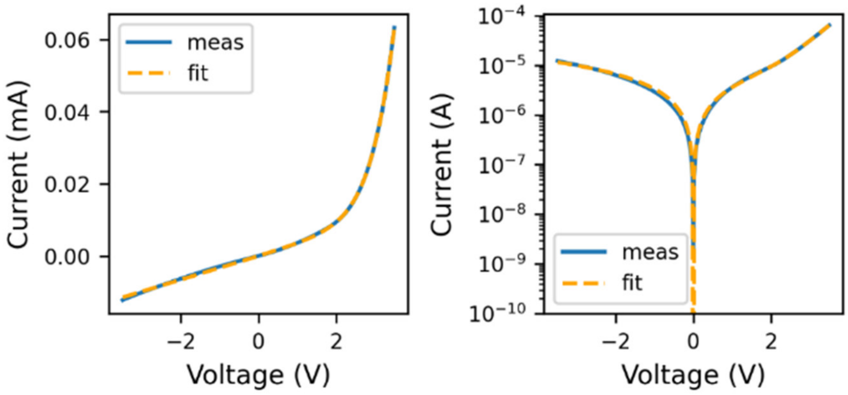

Figure S2. Result of fitting a measurement against the single diode model in a normal (left) and semi-logarithmic (right) plot.

S3 Parameter Space

The ranges of the investigated process parameters are chosen based on empirical knowledge and limitations of the USP system.

Table S3. Considered parameter ranges for the USP deposition of copper oxides.

Parameter	Minimum	Maximum
$\boldsymbol{N}$ **(1)**	30	150
$\boldsymbol{d}$ **(mm)**	155	205
$\boldsymbol{v}$ **(mm min^{-1})**	100	200
$\boldsymbol{Q}$ **(ml min^{-1})**	0.50	1.50
$\boldsymbol{T}$ **(°C)**	220	340
$\boldsymbol{c}$ **(mM)**	12.5	50.0

S4 Sample Data Overview

Table S4. Process parameters for the USP deposition of copper oxides.

ID	Origin	N (1)	d (mm)	v (mm min^{-1})	Q (ml min^{-1})	T (°C)	c (mM)
**00**	BR	67	200	167	1.00	280	25.0
**01**	LHS	55	169	167	0.97	295	35.0
**02**	LHS	108	170	183	0.75	331	16.3
**03**	LHS	75	204	117	1.27	272	37.7
**04**	LHS	121	197	192	1.10	229	45.8
**05**	LHS	31	160	147	1.49	310	26.3
**06**	LHS	144	159	159	0.83	251	32.3
**07**	LHS	41	193	135	1.15	304	23.8
**08**	LHS	131	189	142	0.57	283	47.0
**09**	LHS	72	184	105	0.92	259	21.3
**10**	LHS	99	176	120	0.67	326	43.0
**11**	LHS	89	182	180	1.32	240	13.3
**12**	PO1	30	205	100	1.50	225	50.0
**13**	PO1	30	155	170	0.51	220	50.0
**14**	PO1	110	200	101	0.99	335	13.8
**15**	PO1	37	173	156	1.08	325	32.9
**16**	PO2	30	155	101	1.50	251	12.5
**17**	PO2	30	155	200	1.50	220	12.9
**18**	PO2	150	155	100	1.50	220	12.5
**19**	PO2	150	155	199	1.50	257	50.0
**20**	PO3	150	205	100	0.51	220	50.0
**21**	PO3	138	163	198	0.55	268	50.0
**22**	PO3	146	156	115	0.63	320	23.8
**23**	PO3	63	160	151	0.97	271	13.8
**24**	PO4	143	203	113	0.92	224	13.5
**25**	PO4	142	164	159	1.22	222	24.4
**26**	PO4	125	178	119	1.40	225	13.5
**27**	PO4	127	204	156	0.70	224	19.2
**28**	ME1	79	205	101	1.35	270	39.4
**29**	ME2	150	160	119	1.49	220	14.2
**30**	MV1	79	205	156	1.35	270	20.0
**31**	MV2	79	205	156	1.35	270	10.0

Table S5. Phase composition and texture parameters from the Rietveld refinement of the XRD measurements.

ID	Cu_2O (wt%)	CuO (wt%)	Cu (wt%)	In_2O_3 (wt%)	G_1 Cu_2O (111)	G_1 CuO (-111)
**00**	37	26	0	37	1.00	0.60
**01**	51	28	0	21	1.00	0.65
**02**	0	64	0	36	1.00	0.76
**03**	76	8	0	15	0.80	0.47
**04**	57	36	0	7	1.00	0.66
**05**	44	26	0	30	1.00	0.51
**06**	93	0	0	7	0.87	1.00
**07**	14	55	0	31	1.00	0.69
**08**	65	19	0	17	1.00	0.55
**09**	78	0	0	22	1.00	1.00
**10**	1	77	0	23	1.00	0.83
**11**	75	0	0	25	0.73	1.00
**12**	67	0	14	19	0.77	1.00
**13**	13	63	0	24	1.00	0.66
**14**	0	61	0	39	1.00	0.75
**15**	17	55	0	29	1.00	0.70
**16**	28	43	0	29	1.00	0.62
**17**	14	50	0	36	1.00	0.59
**18**	55	0	40	5	0.70	1.00
**19**	5	93	0	2	1.00	1.00
**20**	80	0	0	20	0.80	1.00
**21**	91	0	0	9	0.91	1.00
**22**	40	44	0	16	1.00	0.76
**23**	60	10	0	30	1.00	1.00
**24**	23	51	0	26	1.00	0.89
**25**	51	0	42	7	0.69	1.00
**26**	51	31	0	19	1.00	0.57
**27**	48	15	0	37	1.00	0.91
**28**	60	0	27	13	0.85	1.00
**29**	32	0	57	11	0.74	1.00
**30**	67	0	2	31	0.69	1.00
**31**	46	0	0	54	0.69	1.00

G_1: Texture parameter in modified March's function (< 1: platy habit)[9].

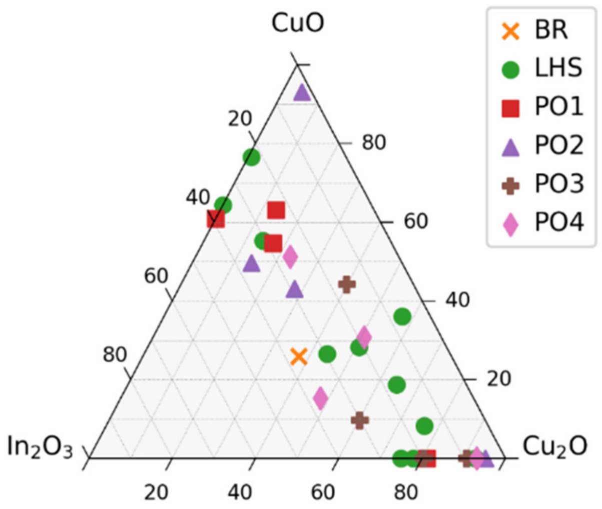

Figure S3. Ternary phase diagram with the data from Table S5, grouped into their process parameter origin. The occasionally measured elemental Cu is added to the amount of Cu_2O.

Table S6. Photovoltaic performance metrics.

ID	$\overline{V_{oc}^{8x8}}$ (V)	$V_{oc,max}^{8x8}$ (V)	$\overline{V_{oc}^{6x6}}$ (V)	$V_{oc,max}^{6x6}$ (V)	FoM (V)	$\overline{J_{sc}^{8x8}}$ (mA/cm²)	$J_{sc,min}^{8x8}$ (mA/cm²)
**00**	0.288	0.469	0.324	0.469	0.547	-2.74E-01	-6.81E-01
**01**	0.233	0.322	0.241	0.320	0.423	-3.14E-01	-4.06E-01
**02**	0.005	0.011	0.006	0.010	0.009	-2.57E-03	-6.60E-02
**03**	0.487	0.610	0.536	0.610	1.007	-9.64E-01	-1.21E+00
**04**	0.335	0.437	0.380	0.437	0.710	-5.88E-02	-8.28E-02
**05**	0.319	0.437	0.345	0.437	0.617	-3.17E-01	-4.11E-01
**06**	0.238	0.296	0.251	0.296	0.464	-1.01E-01	-1.22E-01
**07**	0.236	0.492	0.220	0.382	0.346	-8.85E-02	-1.13E-01
**08**	0.158	0.229	0.170	0.229	0.295	-2.29E-01	-2.78E-01
**09**	0.444	0.555	0.479	0.555	0.893	-7.70E-01	-1.04E+00
**10**	0.002	0.004	0.002	0.003	0.003	-6.06E-03	-1.49E-02
**11**	0.517	0.783	0.547	0.783	0.928	-4.56E-01	-5.65E-01
**12**	0.477	0.860	0.557	0.860	0.917	-3.11E-03	-5.52E-03
**13**	0.075	0.146	0.085	0.146	0.135	-6.64E-03	-1.02E-02
**14**	–	–	–	–	–	–	–
**15**	0.003	0.008	0.003	0.008	0.004	-4.70E-03	-9.76E-03
**16**	0.025	0.062	0.027	0.062	0.040	-5.05E-02	-2.65E-01
**17**	0.212	0.339	0.243	0.339	0.417	-9.00E-03	-6.05E-02
**18**	0.804	0.856	0.815	0.840	1.605	-1.13E-02	-2.24E-02
**19**	0.074	0.107	0.084	0.107	0.150	-5.80E-02	-8.45E-02
**20**	0.324	0.519	0.322	0.464	0.546	-8.92E-02	-1.81E-01
**21**	0.025	0.040	0.029	0.040	0.049	-6.61E-02	-1.05E-01
**22**	0.026	0.044	0.027	0.043	0.045	-2.91E-02	-8.97E-02
**23**	0.111	0.171	0.117	0.171	0.196	-7.83E-02	-1.01E-01
**24**	0.070	0.109	0.074	0.105	0.126	-3.23E-02	-5.01E-02
**25**	0.724	0.867	0.757	0.867	1.417	-2.09E-03	-4.38E-03
**26**	0.150	0.240	0.166	0.240	0.280	-6.43E-02	-8.25E-02
**27**	0.246	0.450	0.280	0.450	0.455	-1.23E-01	-1.67E-01
**28**	0.816	0.999	0.818	0.999	1.489	-2.83E-03	-4.28E-03
**29**	–	–	–	–	–	–	–
**30**	0.331	0.494	0.366	0.494	0.636	-1.06E-02	-1.85E-02
**31**	0.509	0.702	0.579	0.702	1.057	-2.33E-02	-3.41E-02

S5 Effect of GP Target Selection

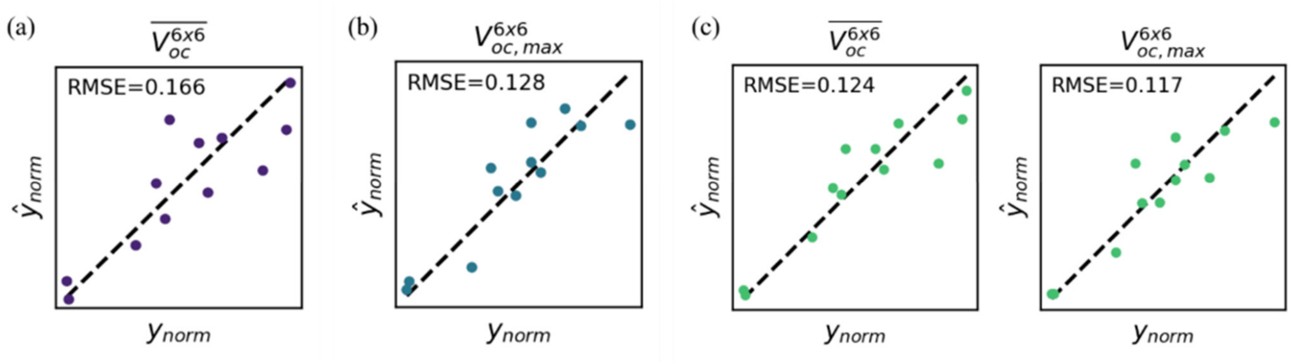

Figure S4. LOOCV of the surrogate model trained on the initial dataset for the prediction of (a) the average and (b) the maximum V_{oc} over the 6 × 6 inner array of devices, and (c) the multitask prediction of both values simultaneously.

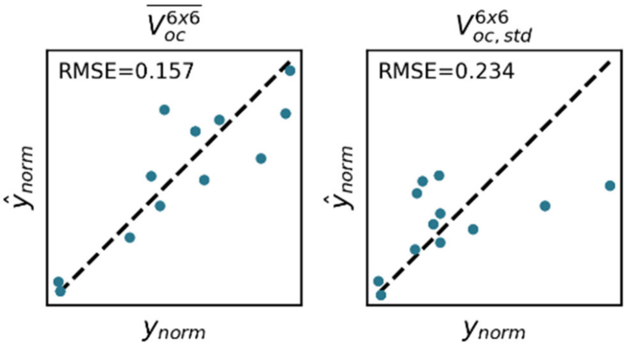

Figure S5. LOOCV of the surrogate model trained on the initial dataset for the multitask prediction of the average V_{oc} and its standard deviation over the 6 × 6 inner array of devices.

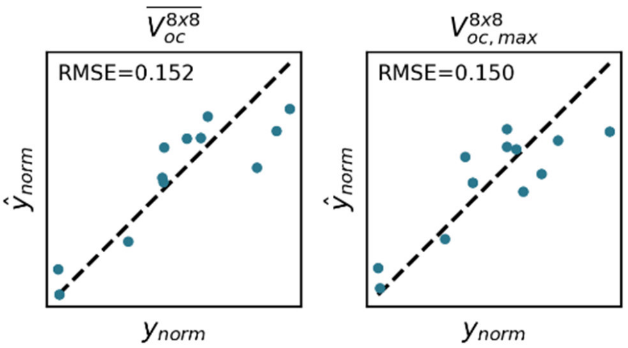

Figure S6. LOOCV of the surrogate model trained on the initial dataset for the multitask prediction of the average and the maximum V_{oc} over the 8 × 8 array of devices.

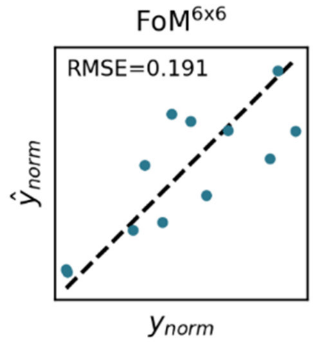

Figure S7. LOOCV of the surrogate model trained on the initial dataset for the prediction of the FoM in the 6 × 6 inner array of devices.

S6 Model Degradation during PO

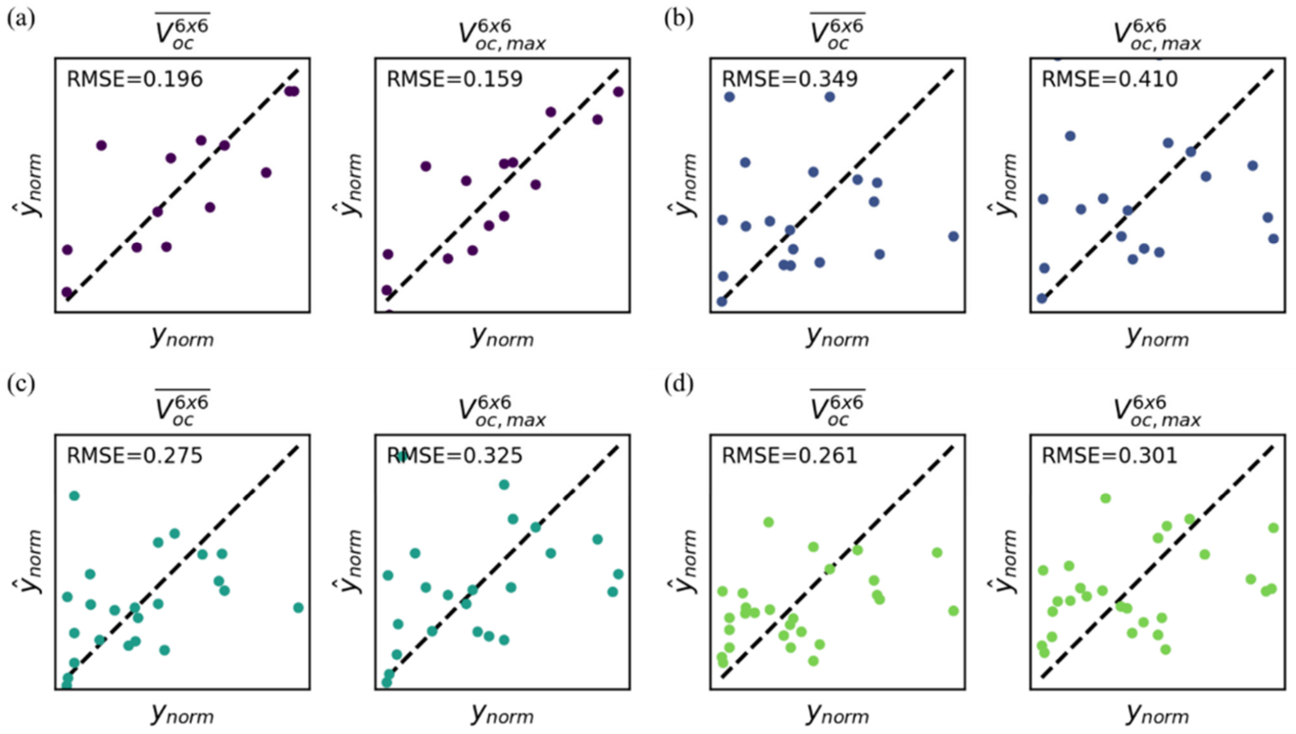

Figure S8. LOOCV of the surrogate model for the multitask prediction of the average and the maximum V_{oc} over the 6 × 6 inner array of devices, trained during the parameter optimization on an increasing number of datapoints through including samples from (a) PO1, (b) PO2, (c) PO3, and (d) PO4.

S7 Results of Model Optimization

Table S7. Sample selection which yields the LOOCV with the lowest RMSE during the model optimization in 2 runs for each MNS of 10, 15, and 20 samples.

ID	MNS10_1	MNS10_2	MNS15_1	MNS15_2	MNS20_1	MNS20_2
**00**	•	–	•	•	•	•
**01**	–	–	–	–	•	•
**02**	•	–	•	•	•	•
**03**	•	•	•	•	•	•
**04**	•	•	•	•	•	•
**05**	–	–	–	–	•	•
**06**	•	•	•	•	•	•
**07**	•	–	•	•	–	–
**08**	–	•	•	•	–	•
**09**	–	•	–	–	•	•
**10**	•	•	•	–	•	•
**11**	–	–	–	–	–	•
**12**	–	–	–	•	–	–
**13**	•	–	•	•	•	•
**14**	–	–	–	–	–	–
**15**	•	•	•	•	•	•
**16**	–	–	•	•	•	•
**17**	–	•	•	•	•	•
**18**	•	•	•	•	•	•
**19**	–	•	–	–	•	•
**20**	–	•	•	•	•	•
**21**	•	•	•	•	•	–
**22**	•	•	•	•	•	•
**23**	•	–	•	•	•	•
**24**	•	–	–	–	•	–
**25**	•	–	•	•	•	•
**26**	–	–	–	–	•	–
**27**	–	–	•	•	•	•
**Σ:**	15	13	19	20	23	22
**RMSE:**	0.138	0.146	0.134	0.146	0.235	0.157

•: Selected. **-:** Not selected. The highlighted column corresponds to the best run.

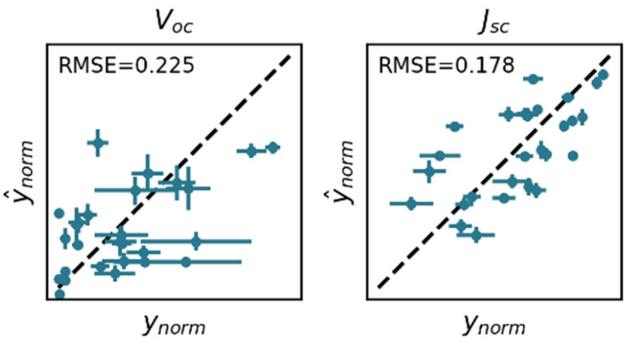

Figure S9. LOOCV of the surrogate model trained on the complete data after the parameter optimization for the multitask prediction of the V_{oc} and J_{sc} of individual datapoints.

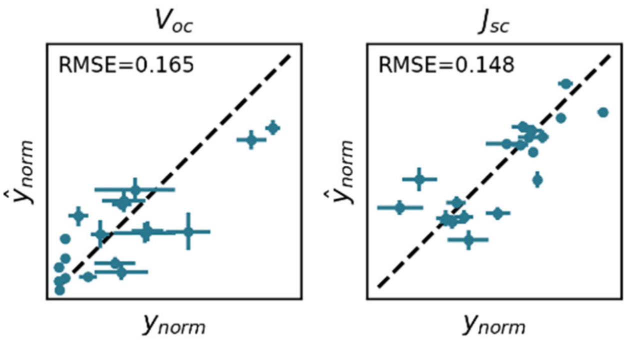

Figure S10. LOOCV of the surrogate model trained on the optimized dataset after the model optimization for the multitask prediction of the V_{oc} and J_{sc} of individual datapoints.

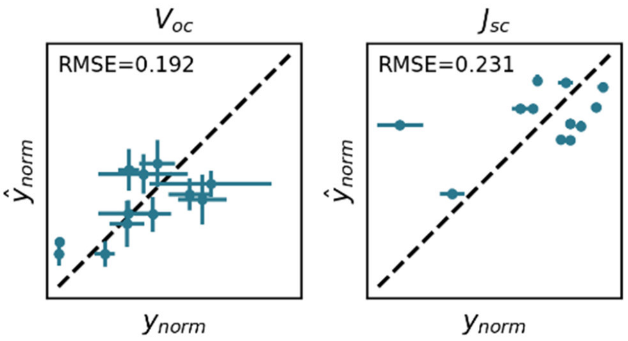

Figure S11. LOOCV of the surrogate model trained on the initial dataset for the multitask prediction of the V_{oc} and J_{sc} of individual datapoints.

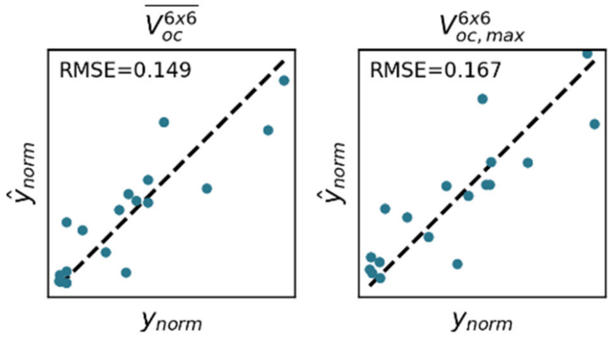

Figure S12. LOOCV of the surrogate model trained on the optimized dataset after the model optimization for the multitask prediction of the average and the maximum V_{oc} over the 6 × 6 array of devices.

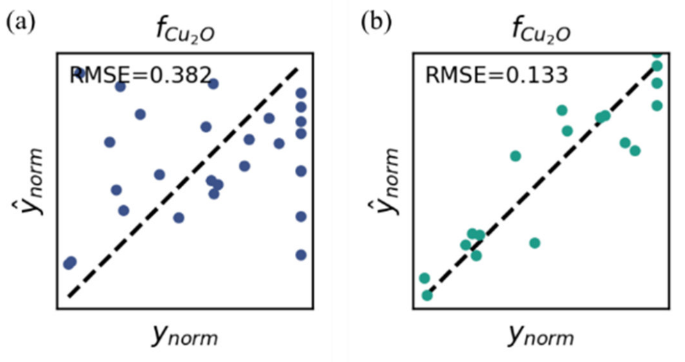

Figure S13. LOOCV of the surrogate model trained on (a) the complete dataset after the parameter optimization and (b) the optimized dataset after the model optimization for the prediction of the Cu_2O fraction.

S8 Results of Model Evaluation

Table S8. Extrema of the average V_{oc} over the 8 × 8 device array after model optimization.

Name	N (1)	d (mm)	v (mm/min)	Q (ml/min)	T (°C)	c (mmol/l)	Occ. (1)	$\overline{V_{oc}^{8x8}}$ (V)
$V_{oc}^{t}(MO)$	72	170	159	0.68	297	39.6	30	-0.005
$V_{oc}^{p}(MO)$	72	205	100	1.49	271	41.4	19	0.503
$V_{oc}^{p'}(MO)$	150	160	115	1.49	220	21.3	11	0.810

Occ.: Occurrences out of 30 optimization runs.

Table S9. Extrema of the average J_{sc} over the 8 × 8 device array after model optimization.

Name	N (1)	d (mm)	v (mm/min)	Q (ml/min)	T (°C)	c (mmol/l)	Occ. (1)	$\overline{J_{sc}^{8x8}}$ (mA/cm²)
$J_{sc}^{t}(MO)$	105	172	200	0.75	336	15.9	18	6.89E-04
$J_{sc}^{t'}(MO)$	147	161	199	0.99	220	43.3	12	1.15E-03
$J_{sc}^{p}(MO)$	85	205	100	1.28	270	39.0	30	1.04E+00

Occ.: Occurrences out of 30 optimization runs.

Table S10. Extrema of the average V_{oc} over the 8 × 8 device array in the initial dataset.

Name	N (1)	d (mm)	v (mm/min)	Q (ml/min)	T (°C)	c (mmol/l)	Occ. (1)	$\overline{V_{oc}^{8x8}}$ (V)
$V_{oc}^{t}(Initial)$	150	178	198	0.50	340	46.3	26	-0.098
$V_{oc}^{t'}(Initial)$	150	155	199	0.50	220	50.0	4	0.129
$V_{oc}^{p}(Initial)$	40	197	101	1.50	250	27.6	30	0.610

Occ.: Occurrences out of 30 optimization runs.

Table S11. Extrema of the average J_{sc} over the 8 × 8 device array in the initial dataset.

Name	N (1)	d (mm)	v (mm/min)	Q (ml/min)	T (°C)	c (mmol/l)	Occ. (1)	$\overline{J_{sc}^{8x8}}$ (mA/cm²)
$J_{sc}^{t}(Initial)$	148	197	197	0.50	340	12.6	19	3.01E-04
$J_{sc}^{t'}(Initial)$	150	155	200	0.50	220	50.0	11	1.42E-02
$J_{sc}^{p}(Initial)$	30	155	100	1.50	279	45.4	30	2.72E+00

Occ.: Occurrences out of 30 optimization runs.

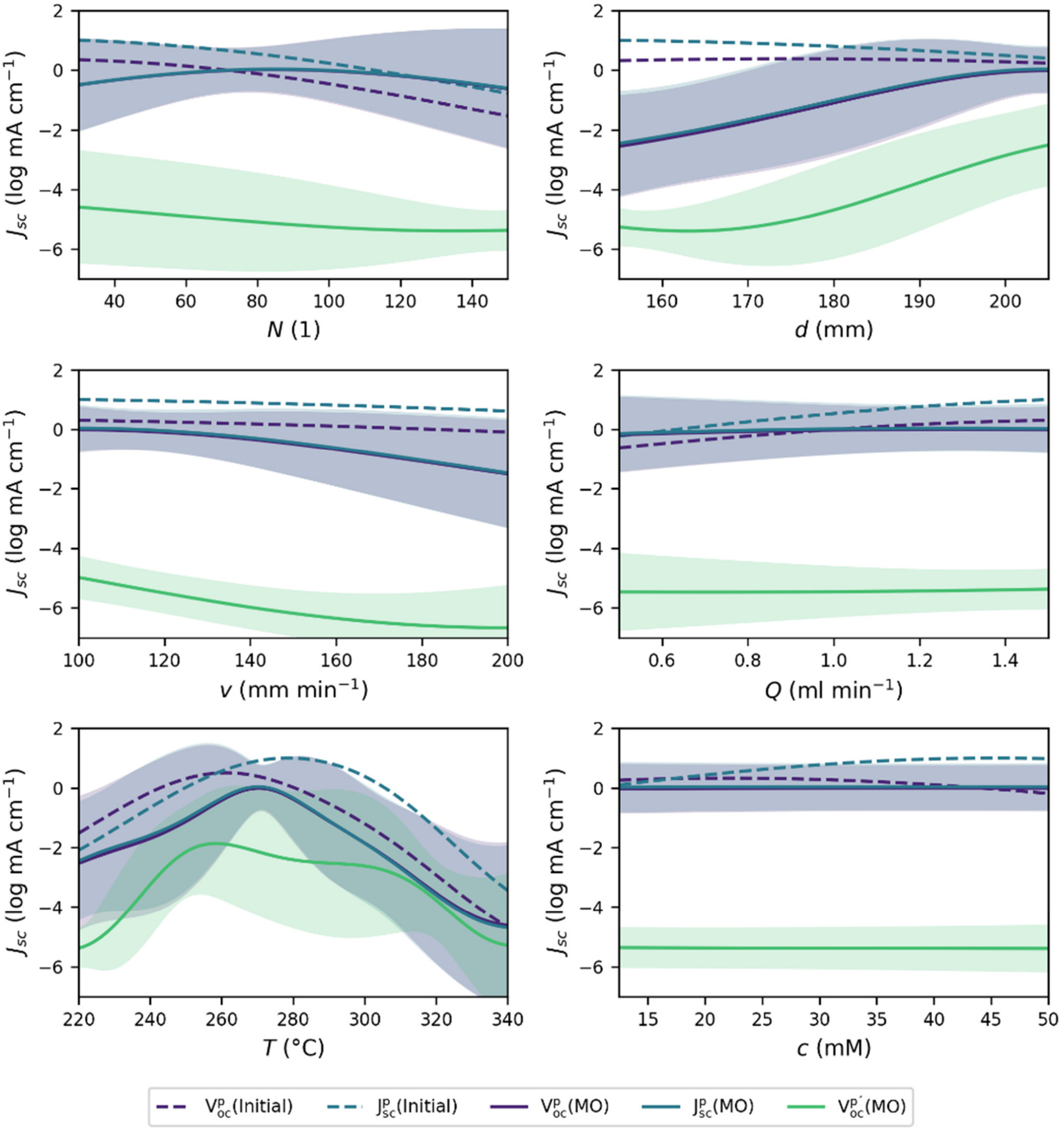

Figure S14. Predicted process parameter dependencies on the J_{sc} corresponding to the sets of parameters which maximize the V_{oc} and J_{sc} in the models trained on the initial dataset and after the model optimization. The shaded areas are the confidence regions of the respective predictions.

S9 Problem with Nebulization

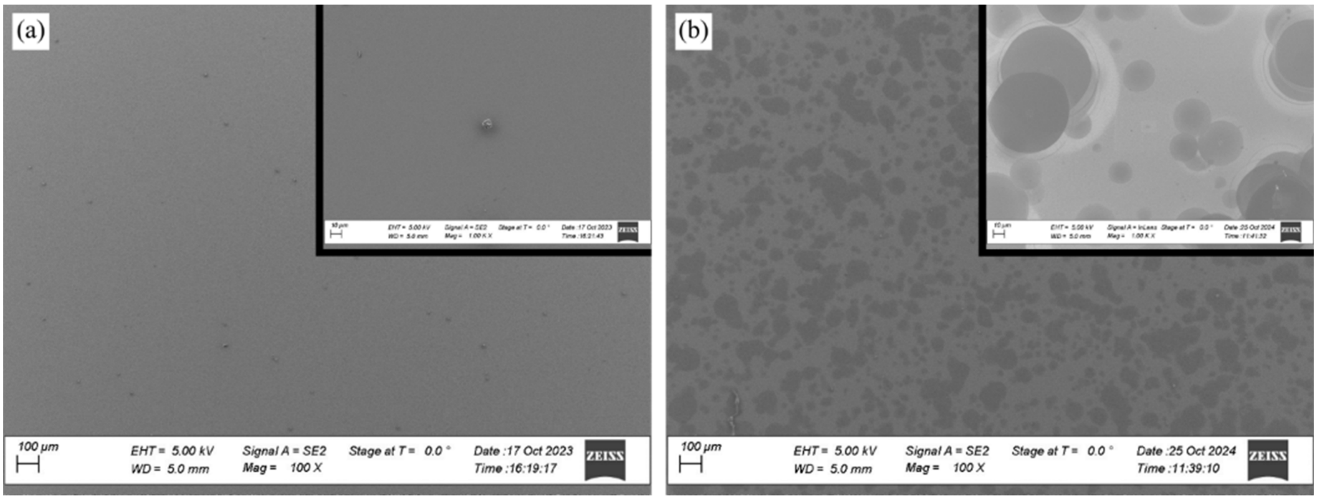

Figure S15. Microstructure of the USP-deposited Ga_2O_3 which is used in this work, recorded by scanning electron microscopy, the insets show the same film at 10x the magnification. (a) Initially, a homogenous film with a few particles scattered around the surface is observed. (b) After the hypothesized nozzle failure, a lot of speckles are visible which appear like impacted droplets at higher magnification.

S10 Maps of Open Circuit Voltage

Table S12. V_{oc} maps of all samples which show the measured open circuit voltage for each individual datapoint. The color palette is fixed to a range of [0, 1] V and the center value in the scale bar denotes the average V_{oc}.

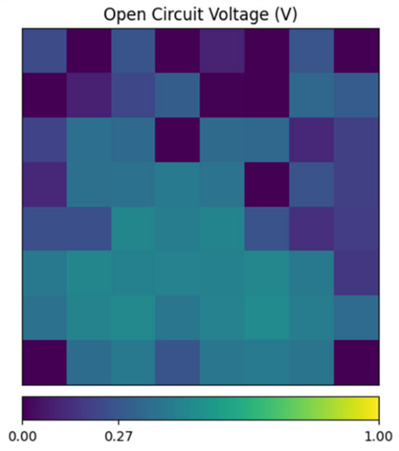

00

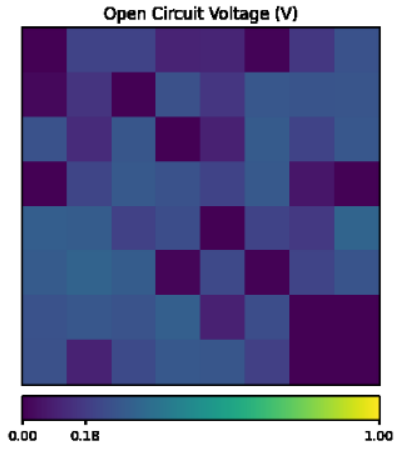

01

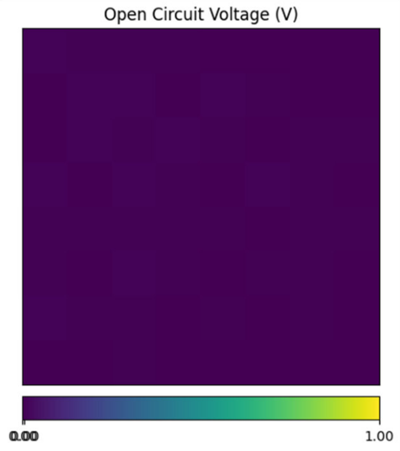

02

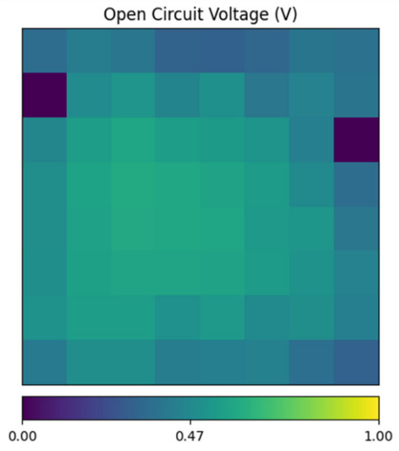

03

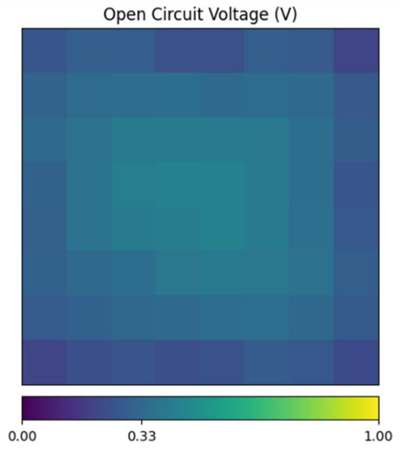

04

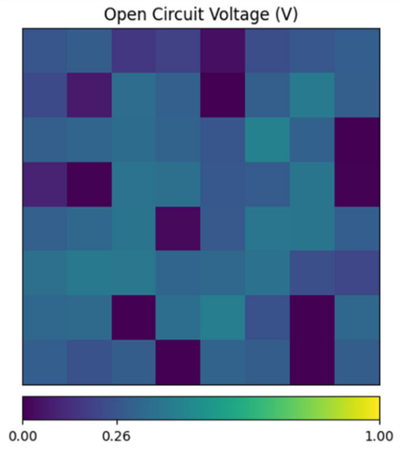

05

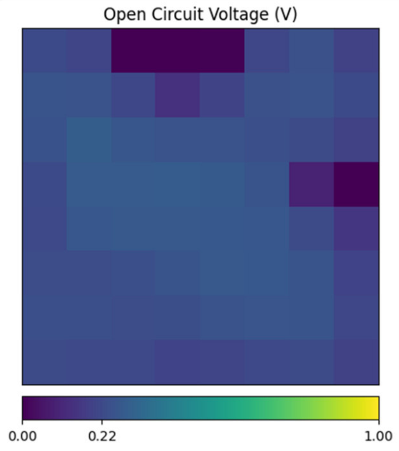

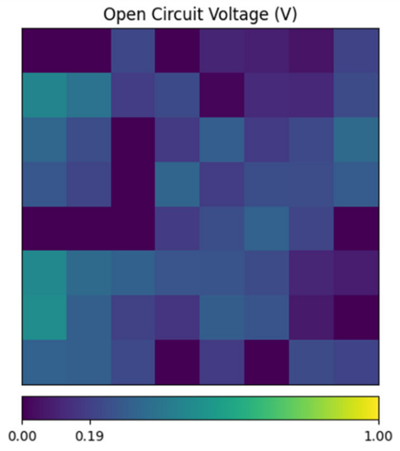

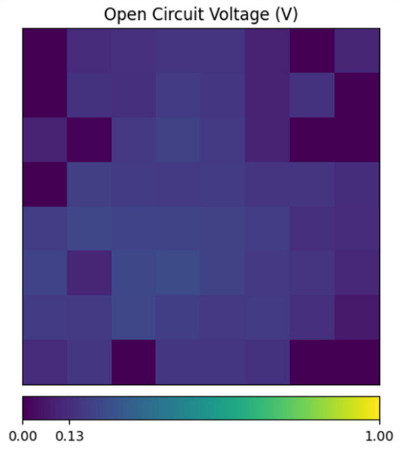

06

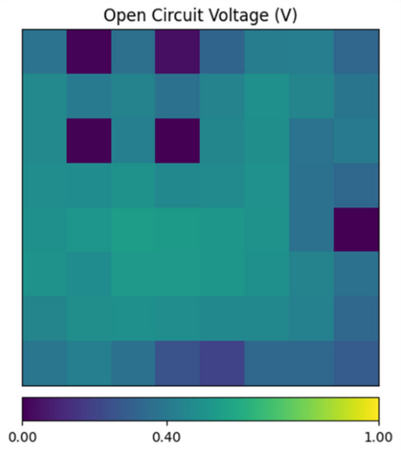

07

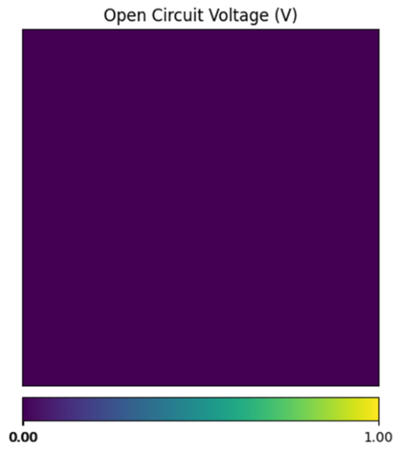

08

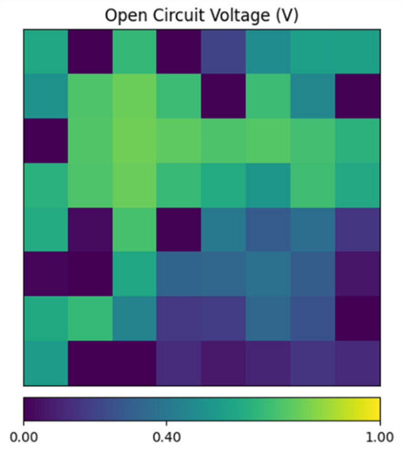

09

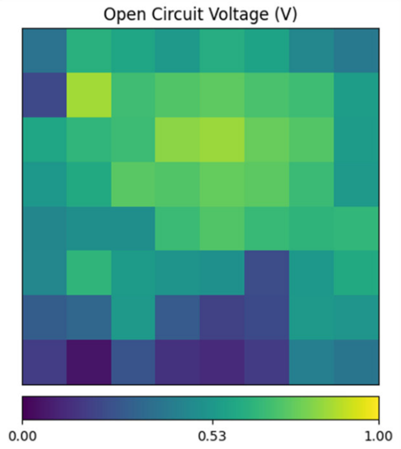

10

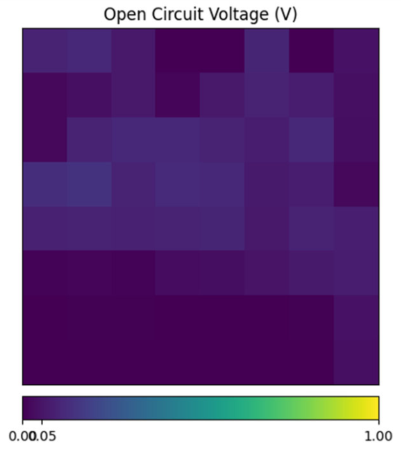

11

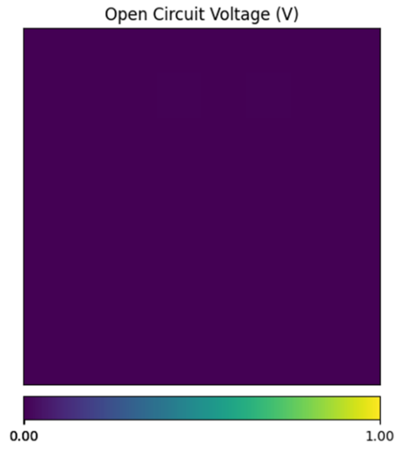

12

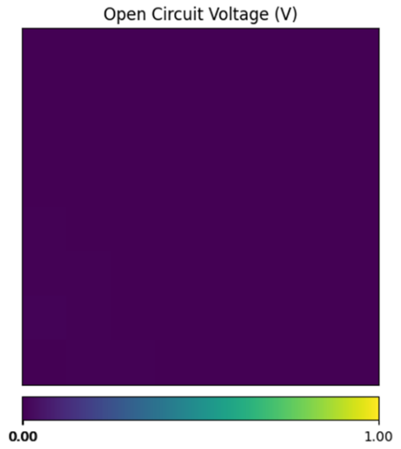

13

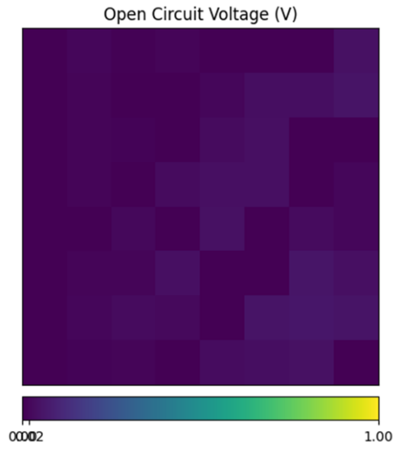

14

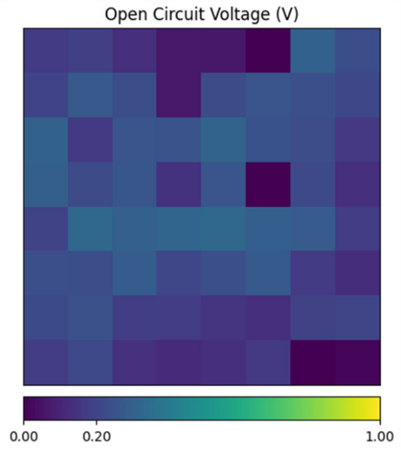

15

16

17

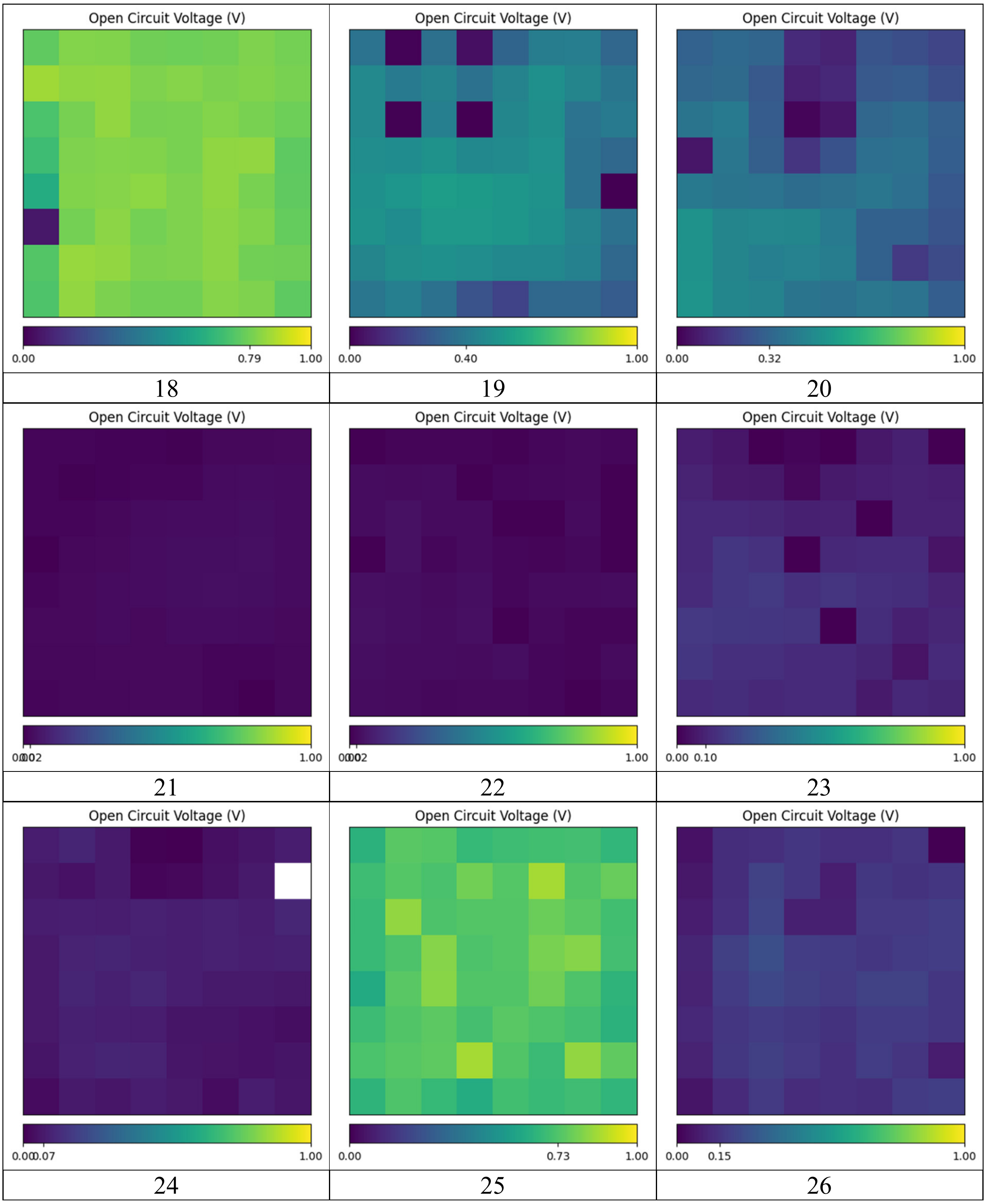
Open Circuit Voltage (V)
0.00
0.79
1.00
18
Open Circuit Voltage (V)
0.00
0.40
1.00
19
Open Circuit Voltage (V)
0.00
0.32
1.00
20
Open Circuit Voltage (V)
0.00
0.02
1.00
21
Open Circuit Voltage (V)
0.00
0.02
1.00
22
Open Circuit Voltage (V)
0.00
0.10
1.00
23
Open Circuit Voltage (V)
0.00
0.07
1.00
24
Open Circuit Voltage (V)
0.00
0.73
1.00
25
Open Circuit Voltage (V)
0.00
0.15
1.00
26

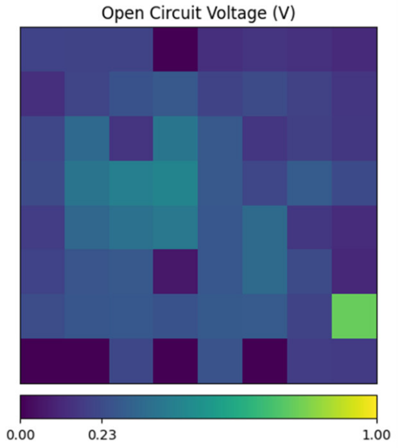Open Circuit Voltage (V)<br>0.00<br>0.23<br>1.00	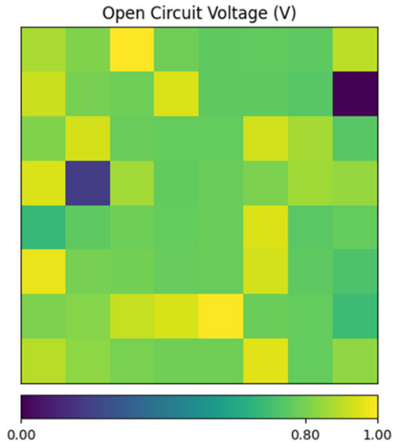Open Circuit Voltage (V)<br>0.00<br>0.80<br>1.00	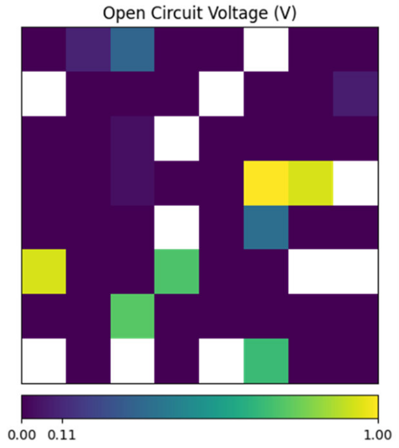Open Circuit Voltage (V)<br>0.00<br>0.11<br>1.00
27	28	29
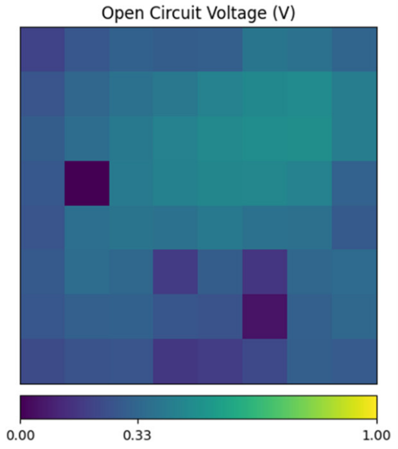Open Circuit Voltage (V)<br>0.00<br>0.33<br>1.00	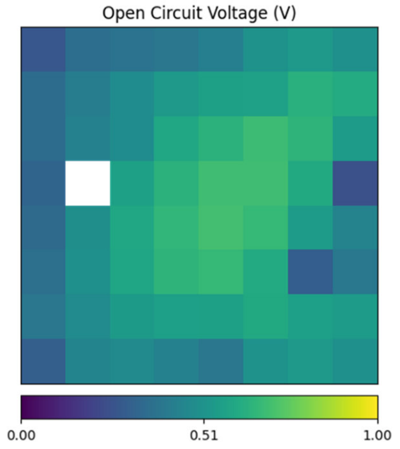Open Circuit Voltage (V)<br>0.00<br>0.51<br>1.00	
30	31	

References

(1) Gražulis, S.; Daškevič, A.; Merkys, A.; Chateigner, D.; Lutterotti, L.; Quirós, M.; Serebryanaya, N. R.; Moeck, P.; Downs, R. T.; Le Bail, A. Crystallography Open Database (COD): An Open-Access Collection of Crystal Structures and Platform for World-Wide Collaboration. *Nucleic Acids Research* **2012**, *40* (D1), D420–D427. https://doi.org/10.1093/nar/gkr900.

(2) Rodríguez-Carvajal, J. FullProf. *CEA/Saclay, France* **2001**, *1045*, 132–146.

(3) Putz, H.; Brandenburg, K. Match!–Phase Analysis Using Powder Diffraction, Crystal Impact. *GbR, Kreuzherrenstr* **2016**, *102*, 53227.

(4) Osorio-Rivera, D.; Torres-Delgado, G.; Márquez-Marín, J.; Castanedo-Pérez, R.; Aguilar-Frutis, M. A.; Zelaya-Ángel, O. Cuprous Oxide Thin Films Obtained by Spray-Pyrolysis Technique. *J Mater Sci: Mater Electron* **2018**, *29* (1), 851–857. https://doi.org/10.1007/s10854-017-7980-5.

(5) Plankensteiner, N.; Kautek, W.; Dimopoulos, T. Aqueous Spray Pyrolysis of Cu2O Films: Influence of Reducing Agent and Acetic Acid Addition. *ChemNanoMat* **2020**, *6* (4), 663–671. https://doi.org/10.1002/cnma.202000006.

(6) Moumen, A.; Hartiti, B.; Thevenin, P.; Siadat, M. Synthesis and Characterization of CuO Thin Films Grown by Chemical Spray Pyrolysis. *Opt Quant Electron* **2017**, *49* (2), 70. https://doi.org/10.1007/s11082-017-0910-1.

(7) Prakash, A.; V s, G. K.; Moger, S. N.; Mahesha, M. G. Spectroscopic and Electrical Analysis of Spray Deposited Copper Oxide Thin Films. *Materials Today Communications* **2022**, *32*, 103926. https://doi.org/10.1016/j.mtcomm.2022.103926.

(8) Jain, A.; Kapoor, A. Exact Analytical Solutions of the Parameters of Real Solar Cells Using Lambert *W*-Function. *Solar Energy Materials and Solar Cells* **2004**, *81* (2), 269–277. https://doi.org/10.1016/j.solmat.2003.11.018.
